# TRPA1 Expression and Pathophysiology in Immune Cells

**DOI:** 10.3390/ijms222111460

**Published:** 2021-10-24

**Authors:** Robbe Naert, Alejandro López-Requena, Karel Talavera

**Affiliations:** 1 Laboratory of Ion Channel Research, Department of Cellular and Molecular Medicine, KU Leuven, VIB Center for Brain & Disease Research, 3000 Leuven, Belgium; robbe.naert@kuleuven.be (R.N.); alejandro.lopezrequena@sanofi.com (A.L.-R.); 2 Ablynx, Technologiepark 21, 9052 Zwijnaarde, Belgium

**Keywords:** TRPA1, eosinophils, basophils, macrophages, mast cells, NK cells, dendritic cells, T cells, B cells, immunity

## Abstract

The non-selective cation channel TRPA1 is best known as a broadly-tuned sensor expressed in nociceptive neurons, where it plays key functions in chemo-, thermo-, and mechano-sensing. However, in this review we illustrate how this channel is expressed also in cells of the immune system. TRPA1 has been detected, mainly with biochemical techniques, in eosinophils, mast cells, macrophages, dendritic cells, T cells, and B cells, but not in neutrophils. Functional measurements, in contrast, remain very scarce. No studies have been reported in basophils and NK cells. TRPA1 in immune cells has been linked to arthritis (neutrophils), anaphylaxis and atopic dermatitis (mast cells), atherosclerosis, renal injury, cardiac hypertrophy and inflammatory bowel disease (macrophages), and colitis (T cells). The contribution of TRPA1 to immunity is dual: as detector of cell stress, tissue injury, and exogenous noxious stimuli it leads to defensive responses, but in conditions of aberrant regulation it contributes to the exacerbation of inflammatory conditions. Future studies should aim at characterizing the functional properties of TRPA1 in immune cells, an essential step in understanding its roles in inflammation and its potential as therapeutic target.

## 1. Introduction

**Figure 1:**
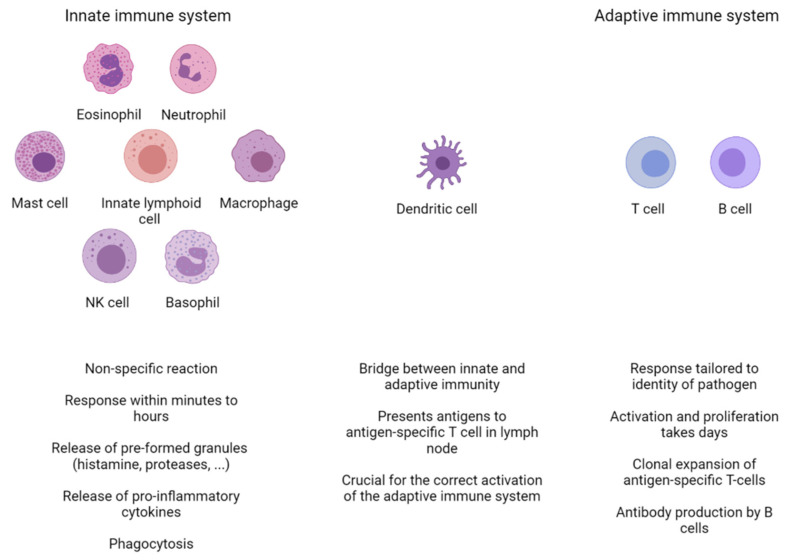
Overview of the different cell types of the innate and adaptive immune systems. The innate immune system is the first line of defense. It consists of eosinophils, neutrophils, macrophages, basophils, natural killer (NK) cells, mast cells, and innate lymphoid cells. They are capable of responding within minutes to foreign pathogens through a variety of non-specific mechanisms, including the release of granules and pro-inflammatory cytokines. In some cases, this initial response is followed by a reaction from the adaptive immune system, consisting of T and B cells. The adaptive immune response takes longer to develop and is crucially dependent on dendritic cells (DCs). DCs patrol their microenvironment, take up and process antigen material, and present it to T cells in the local lymph node. This results in the clonal expansion of an antigen-specific T cells, which will recirculate to the site of infection and help clear up the infection. Activated B cells will proliferate and differentiate into antibody-secreting plasma cells.

The immune system is a highly adaptable and versatile system capable of recognizing a wide range of pathogens, ranging from the smallest virus to the largest tapeworm. It consists of two interconnected branches: the innate and the adaptive immune system. The innate immune system is the first line of defense and is responsible for the fast, non-specific, initial immune response. In addition to the defense mechanisms relying on barrier and clearance functions, which are mainly based on the activities of epithelial cells, the innate immunity is based on multiple cell types, including eosinophils, neutrophils, mast cells, innate lymphoid cells, macrophages, natural killer (NK) cells, and basophils (Figure 1). Sometimes, the innate immune system is capable of eliminating the pathogen by itself, but in other cases the adaptive immune system steps in. Dendritic cells link the two systems by detecting antigens and presenting them to T cells. The adaptive immune system, based on T and B cells, has a slower activation mechanism (5–6 days), but has a more targeted approach, tailored by the identity of the intruder. The proper activation and propagation of an immune reaction requires a balance between pro- and anti-inflammatory mediators. Disruption of this balance leads to the survival of the pathogen and/or the destruction of collateral healthy tissue.

Central to these responses are the multiple functions of immune cells, which in turn rely heavily on Ca^2+^-signaling pathways. For instance, antigen recognition via the T cell receptor results in the IP_3_-dependent release of Ca^2+^ from the endoplasmic reticulum (ER). This decrease in ER Ca^2+^ levels leads to the activation of Ca^2+^-release-activated Ca^2+^ channels (CRAC) in the membrane and the influx of extracellular Ca^2+^. The resulting increased level of intracellular Ca^2+^ activates Ca^2+^-dependent enzymes and downstream transcription factors such as NF-κB and NFAT, which further steer the activation and differentiation of T cells [1]. This crucial role for Ca^2+^ also extends to other immune cells such as- B cells [2], macrophages [3], and other members of the innate immune system [4].

Immune cells express many players regulating intracellular Ca^2+^ homeostasis such as CRAC channels and purinergic receptors. Among this group of Ca^2+^ regulators, the role and importance of transient receptor potential (TRP) channels has become increasingly clear. This family of cation channels consists of 28 mammalian members and is divided into six groups based on sequence homology: TRP canonical (TRPC), TRP vanilloid (TRPV), TRP melastatin (TRPM), TRP polycystin (TRPP), TRP mucolipin (TRPML), and TRP ankyrin (TRPA). All TRP proteins consist of six putative transmembrane domains with a pore region between the fifth and the sixth domain. In accordance with their role as polymodal sensors, TRP channels are activated by a wide variety of stimuli including osmotic stress, pressure, temperature changes, and endogenous and exogenous chemicals including endogenous inflammatory mediators [5]. Combined with their widespread expression patterns, these channels regulate many physiological processes in multiple cell types, including immune cells such as dendritic cells [6,7,8] and T cells [9,10].

This review focuses on the sole member of the mammalian TRPA family, TRPA1, a non-selective cation channel originally described in human fibroblasts [11,12,13]. Since then, the pattern of TRPA1 has been found to extend significantly to a subpopulation of sensory neurons [14,15], epithelial cells [16], melanocytes [17], and keratinocytes [18]. Functionally, TRPA1 activation leads to cation influx, which leads to cell membrane depolarization and an increase in the intracellular Ca^2+^ concentration [19], which in turn modulates channel activation and desensitization, as well as multiple signaling pathways [13]. It is best known as a broadly-tuned chemo-sensor, due to its activation by a variety of chemicals, including reactive electrophiles such as cinnamaldehyde [20], mustard oils [21], allicin [22], and acrolein [23]; redox agents such as H_2_O_2_ and hypochlorite [24]; gasotransmitters (e.g., nitric oxide and hydrogen sulphide) [25]; and heavy metals (Zn^2+^, Cu^2+^, and Cd^2+^) [26,27,28]. In addition, TRPA1 is activated by a plethora of non-electrophilic compounds, such as menthol [29], nicotine [30], local and general anaesthetics [31,32,33], and bacterial lipopolysaccharides [34,35,36,37]. TRPA1 is regulated by numerous factors, including cholesterol [38,39], changes in pH, Ca^2+^, phosphatidylinositol 4,5-bisphosphate (PIP_2_), phosphatases, protein phosphorylation, and protein-protein interactions [13]. TRPA1 can be activated by cold [40,41,42,43], heat [44], and mechanical stimulation [45,46], including membrane perturbations induced by intercalation of chemical compounds in the lipid bilayer [47,48,49,50,51]. Many of the TRPA1 stimuli function as signals associated with cell damage [52], and the channel can actually integrate the actions of these stimuli, resulting in stronger responses. For instance, both 4-hydroxy-2-nonenal, an endogenous product of lipid peroxidation during inflammation as well as trinitrophenol, which causes membrane curvature, sensitize TRPA1 activation by lipopolysaccharides [34].

The activity of TRPA1 can be inhibited by several natural and synthetic compounds [13], of which HC-030031 [53], AP-18 [54], and A-967079 [55] have been the most widely employed.

Although the function of TRPA1 in sensory neurons has been extensively described, its roles in immune cells has been long overlooked. This review gives an overview of TRPA1 expression and (patho)physiological functions in immune cells. TRPA1 activation in sensory neurons and other cells can also influence the functions of immune cells and modulate immune responses [56], but we considered these interactions to be indirect and therefore we did not include them in this review.

## 2. TRPA1 Expression in Primary and Secondary Lymphoid Organs

TRPA1, at the time known as ANKTM1, was not found by Northern blot in murine spleen [40]. Concomitantly, qPCR analysis of murine spleen also failed to detect TRPA1 [57].

In contrast, low levels were detected in human spleen, thymus, and lymph nodes, although Western blot analysis and immunohistochemistry of the former tissue failed to detect the protein [58]. TRPA1 expression was also detected in human peripheral blood leukocytes by an enzyme-linked immunosorbent assay [59]. Although the application of menthol, a TRPM8/TRPA1 ligand, leads to Ca^2+^-induced cell death in HL-60 cells [60], this effect is most likely independent of TRPA1, as qPCR experiments failed to detect TRPA1 in this cell line [61].

## 3. TRPA1 Expression and Function in Innate Immunity

### 3.1. Eosinophils

#### 3.1.1. Expression

Immunohistochemistry failed to detect TRPA1 in murine skin eosinophils [62] but did show TRPA1 in eosinophils in nasal polyps of chronic rhinosinusitis patients [63].

#### 3.1.2. (Patho)physiology

To date there have been no reports on the function of TRPA1 in eosinophils, most likely due to its low/absent expression.

### 3.2. Neutrophils

#### 3.2.1. Expression

TRPA1 expression levels in resting human neutrophils were very low or non-existent [64], based on nine studies (E-GEOD-8507, E-GEOD-8668, E-GEOD-28491, E-GEOD-16837, E-GEOD-12662, E-GEOD-28490, E-GEOD-22103, E-GEOD-22886, and E-GEOD-2322) logged in the Gene Expression Omnibus [65].

#### 3.2.2. (Patho)physiology

The activation of TRPA1 in neutrophils has been linked to arthritis, although it remains unclear if this is due to a direct role of TRPA1 in neutrophils or an indirect effect of non-immune TRPA1. The application of a sulphide donor, GYY4137, protects against serum-transfer induced arthritis in WT mice but not in *Trpa1^-/-^* mice [66]. Because neutrophils play a crucial role in this disease model [67], this protective effect could be partially mediated by TRPA1 on neutrophils, although the underlying mechanisms remains unclear. In a CFA-induced arthritis model, *Trpa1^-/-^* mice exhibited reduced arthritis symptoms and myeloperoxidase activity, as a reflex of neutrophil activation, in the ankle joints [68].

### 3.3. Basophils

Up until now, there are no studies reporting expression or function of TRPA1 in basophils.

### 3.4. Mast Cells

#### 3.4.1. Expression

TRPA1 was detected with RT-PCR in bone marrow-derived mast cells (BMMCs) generated in vitro [69,70]. Immunohistochemistry [71], Western blot [71], and flow cytometry [62] confirmed the presence of TRPA1 in BMMCs. Protein staining was also detected in infiltrating mast cells of murine skins lesions [62]. Interestingly, one research group showed functional expression of TRPA1 in BMMCs [72], whereas another group failed to do so [73]. Allyl isothiocyanate (AITC) induced Ca^2+^ responses in the murine mast cell line C57.1 [62], and Western blot and immunohistochemistry showed TRPA1 in the RBL2H3 rat mast cell line [74].

TRPA1 expression in human mast cells seems to be influenced by tissue localization and inflammatory conditions. TRPA1 gene transcripts were present in human mast cells derived from CD34^+^-peripheral blood progenitor cells via RT-PCR [69]. Microarray [75] and flow cytometry [76] experiments did not detect any TRPA1 in human lung mast cells. However, immunohistochemistry revealed TRPA1 expression in human mast cells of skin lesions [62] and nasal polyps [63]. In contrast to rodent mast cell lines, the human mast cell lines HMC-1 [76] and LAD2 [75] lacked detectable *Trpa1* mRNA expression and only very weak TRPA1 staining was seen in the former [76].

#### 3.4.2. (Patho)physiology

Mast cells exert most, if not all, of their functions through the release of intracellular granules in a process called degranulation. Early findings indicated a possible role for TRPA1 in mast cell degranulation. TRPA1 were localized to the plasma membrane and intracellular vesicles in the RBL2H3 mast cell line. Co-immunoprecipitation experiments in HEK293 and RBL2H3 cells show TRPA1 interacting with secretogranin III, an important player of the granule secretory machinery in mast cells [74]. Since then, there have been contradicting results on the role of TRPA1 in mast cell degranulation. The application of thymol, a TRPA1 agonist, induced mast cell degranulation in MC/9 cells, a murine mast cell line [72]. However, incubating peritoneal mast cells with varying concentrations of AITC (1–1000 μM) did not induce histamine release, indicating that either peritoneal mast cells do not express functional TRPA1 or that this channel does not play a role in peritoneal mast cell degranulation [77]. Moreover, the application of cinnamaldehyde resulted in the same level of mast cell degranulation in both WT and *Trpa1^-/-^* BMMCs, indicating a TRPA1-independent effect on degranulation [78].

Anaphylaxis is a severe allergic reaction partly caused by a massive degranulation of mast cells. The release of huge amounts of preformed mediators such as histamine, tryptase, chymase, and proteoglycans can lead to rapid onset of flushing, swelling, shortness of breath, and in severe cases death [79]. TRPA1 might be a target in the treatment of anaphylaxis. The therapeutic potential of TRPA1 was investigated in a passive cutaneous anaphylaxis (PCA) model. This model consists of sensitization with anti-DNP-IgE, followed by a challenge with DNP-HSA. The application of thymol, a TRPA1 agonist, in between the sensitization and challenge phases attenuated the early and late-phase responses. Thymol induced Ca^2+^ responses in BMMCs, which could be partially blocked by HC-030031. Treating MC/9 cells with thymol also increased Il-6 and Il-13 expression levels but, surprisingly, this effect was not visible at protein level. This discrepancy was caused by thymol-induced apoptosis of BMMCs. These results indicate that thymol could attenuate anaphylaxis by inducing TRPA1-dependent apoptosis in mast cells [72]. The protective role of TRPA1 in anaphylaxis was later questioned in a different animal anaphylaxis model, oxygen-induced anaphylaxis (OIA). The rapid shift of hyperoxia to normoxia (relative hypoxic stress) leads to systemic anaphylaxis, characterized by hypothermia, increased vascular permeability, and damage to the blood-brain barrier. These effects were absent in mast cell-deficient (*Cpa3^Cre^*), *Trpa1^-/-^* and double *Cpa3^cre^; Trpa1^-/-^* animals. The i.p. injection of WT BMMCs into *Cpa3^Cre^* mice successfully “rescued” the phenotype, whereas the injection of *Trpa1^-/-^* BMMCs failed to do so. *Trpa1^-/-^* animals, exposed to hypoxic shift, also showed intact mast cells in the dorsal skin compared to degranulated mast cells in WT animals, an effect that was also seen in WT and *Trpa1^-/-^* BMMCs in vitro. Moreover, relative hypoxic stress resulted in mast cell degranulation and higher levels of mast cell tryptase and histamine in WT BMMC supernatant compared to *Trpa1^-/-^*. Similarly, relative hypoxic stress induced increased tryptase β 2 levels in LAD2 supernatant, but not in the presence of the TRPA1 antagonist HC-030031. These results highlight a TRPA1-induced mast cell-dependent mechanism responsible for the development of oxygen-induced anaphylaxis [71]. The discrepancy between these two studies might be explained by the fact that PCA is an IgE-dependent anaphylaxis model, whereas OIA is IgE-independent.

Intense itch is one of the major symptoms affecting the quality of life of atopic dermatitis patients [80]. In an IL-13-transgenic mouse model of this disease, a TRPA1 antagonist reduced the itch scratching behaviour, indicating a role for this channel in pruritogenesis. TRPA1 expression levels were higher in murine and human skin lesions, specifically in afferent neurons and mast cells. In contrast, TRPA1 was barely detected in other immune cell populations such as eosinophils, Langerhans cells, macrophages, and T cells. The genetic deletion of mast cells had the same attenuating effect on scratching as a TRPA1 antagonist [62].

### 3.5. NK Cells

Up until now, there have been no studies published on the expression or function of TRPA1 in NK cells.

### 3.6. Innate Lymphoid Cells (ILCs)

Up until now, there have been no studies published on the expression or function of TRPA1 in ILCs

### 3.7. Macrophages

#### 3.7.1. Expression

TRPA1 was absent in naïve and activated murine peritoneal macrophages on the mRNA level [81,82,83] and protein level [83]. These cells also showed no increase in intracellular Ca^2+^ levels upon AITC application [83]. Immunohistochemistry showed minimal TRPA1 expression in murine Langerhans cells [62] and skin macrophages [62], but revealed positive staining in murine interstitial macrophages [84] and infiltrating macrophages of atherosclerotic lesions [85].

Tests with qPCR did not detect TRPA1 in human lung parenchyma, suggesting that the channel is not expressed in human alveolar macrophages [86]. Immunohistochemistry, qPCR, and Western blot detected TRPA1 in human monocytes [87]. In contrast, other groups failed to find TRPA1 expression in human monocytes via qPCR [61,88]. Moreover, TRPA1 transcript were also undetectable in human alveolar macrophages and monocyte-derived macrophages [61]. One group was unable to detect TRPA1 in THP-1- and U937-derived macrophages [61], whereas others show TRPA1 staining [89] and expression [90] in THP-1- and U-937-derived macrophages, respectively. Immunohistochemistry showed positive TRPA1 signal in infiltrating macrophages in human ectopic endometrial tissue [91], inflamed colon of IBD patients [84], human oral submucosa [92], and nasal polyps of chronic rhinosinusitis patients [63]. In contrast to mice, human Langerhans cells stained positive for TRPA1 [58].

#### 3.7.2. (Patho)physiology

TRPA1 activation in monocytes was linked to pro-inflammatory effects such as increased TNFα and decreased Il-10 levels [87]. The effect in macrophages seems to be less clear. Cinnamaldehyde decreased the secretion of the pro-inflammatory Il-1β and TNFα, abrogated ROS release, and inhibited phosphorylation of ERK1/2 and JNK1/2 in activated J774.1 macrophages. Decreased secretion level of Il-1β and TNFα were also seen in human blood-derived macrophages and human THP-1-derived macrophages [93]. In contrast, acrolein, a TRPA1 agonist and component of cigarette smoke, induced the release of TNFα from U-937 derived macrophages and Il-8 release in human alveolar macrophages, THP-1 monocytes, and U-937 derived macrophages [94]. TRPA1 ligands have also been found to reduce nitric oxide production in LPS-stimulated mouse macrophages, including the RAW264.7 cell line [95], J774.1 macrophage cell line [96], and peritoneal macrophages [81].

Because macrophages play key roles in atherosclerosis onset and progression [97], it was investigated if TRPA1 was important in the regulation of macrophage activation and polarization. Atherosclerotic aortas of *ApoE^-/-^* mice showed increased TRPA1 expression levels compared to WT aortas, with enhanced TRPA1 staining in macrophage areas of atherosclerotic lesions. Interestingly, *ApoE^-/-^* mice, treated with HC030031 had larger atherosclerotic areas, increased levels of pro-inflammatory cytokines, and hyperlipidemia. Genetic deletion of TRPA1 resulted in a similar phenotype. Treatment with AITC improved all these parameters in *ApoE^-/-^* but not in *ApoE^-/-^Trpa1^-/-^* mice. Oxidized low-density lipoprotein (ox-LDL), crucial in the atherosclerosis pathogenesis and the formation of macrophage foam cells, directly activated TRPA1 in transfected HEK293 cells, an effect abrogated in the presence of HC030031. Concomitantly, ox-LDL induced TRPA1-dependent Ca^2+^ responses in bone marrow-derived macrophages (BMDMs). Inhibition or deletion of TRPA1 in macrophages increased intracellular lipid accumulation through downregulation of ATP-binding cassette-dependent cholesterol efflux, thereby contributing to macrophage foam cell formation. Thus, TRPA1 activation seems to limits macrophage foam cell formation, thereby attenuating atherosclerotic progression [85].

**Figure 2:**
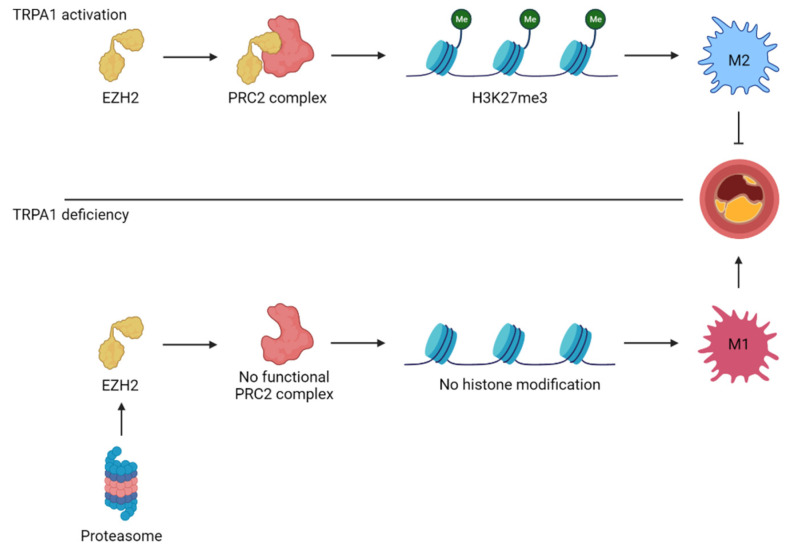
TRPA1 controls the polarization state of macrophages through histone modifications. TRPA1 protects EZH2, one of four subunits of the polycomb repressive complex 2 (PRC2), against proteasomal degradation. The functional PRC2 complex tri-methylates lysine 27 on histone protein 3 (H3K27me3), resulting in closed chromatin and suppression of M1 macrophage-polarizing genes. This shifts the macrophage towards an M2 phenotype and protects against atherosclerotic progression. Contrary to that, TRPA1 deficiency leads to EZH2 degradation and open chromatin. This differentiates macrophages into M1 macrophages and facilitates atherosclerotic progression.

These findings were later confirmed by Wang et al., who further explored the mechanism behind the protective effect of TRPA1 in atherosclerosis. The expression of several M1 markers was increased in *Trpa1^-/-^* BMDMs while the M2 markers were decreased. Moreover, cinnamaldehyde treatment in WT BMDMs had the opposite effect and shifted the polarization profile towards an M2 phenotype. This effect on macrophage polarization was linked to a TRPA1-mediated effect on H3K27me3, an epigenetic modification to histone 3 resulting in a downregulation of M1 macrophage genes. This histone modification is regulated by the polycomb repressive complex 2 (PRC2). Results indicate that TRPA1 protects EZH2, one of the subunits of the PRC2 complex, from protein degradation, thereby allowing PCR2 to induce H3K27me3 histone modification. This modification leads to the suppression of M1 macrophage genes and results in the polarization towards the atherosclerotic-protective M2 macrophages. In contrast, the loss of TRPA1 leads to degradation of EZH2, transcription of M1 macrophage related genes, and atherosclerotic plaque formation [98] (Figure 2).

In sharp contrast with these two studies, TRPA1 seems to exert pro-inflammatory effects in a macrophage cell line in the context of atherosclerosis. The application of lysophosphatidylcholine (LPC), a component of ox-LDL, increased both cytoplasmic and mitochondrial Ca^2+^ levels in THP-1 derived macrophages, but not in the presence of TRPA1 inhibitors. Moreover, inhibition of TRPA1 also reduced several LPC-induced effects including mitochondrial ROS levels and Il-1β secretion [99]. Similar results were described for ATP-induced effects on THP-1 macrophage cell line [89]. The discrepancy surrounding the protective effect of TRPA1 in atherosclerosis might be caused by the use of different macrophage sources (BMDMs vs. THP-1 derived macrophages) and the different stimuli (Ox-LDL vs. LPC) [99].

The anti-inflammatory effect of TRPA1 on macrophages was also seen in an angiotensin II-induced renal injury model. *Trpa1* KO animals showed increased renal injury and increased inflammatory cytokine expression compared to WT animals. There was, however, no difference in blood pressure (BP) between *Trpa1^-/-^* and WT mice. This finding indicates that there are BP-independent effects responsible for this disease phenotype. Because macrophages play a key role in the hypertensive kidney injury model and the amount of M1 and M2 macrophages were increased in kidneys of *Trpa1^-/-^* animals compared to WT, the role of TRPA1 was assessed in RAW 273.4 macrophages. Interestingly, TRPA1 was decreased upon macrophage activation by phorbol-12-myristate-13-acetate (PMA), suggesting that inflammatory conditions might regulate TRPA1 expression. Accordingly, TRPA1 mRNA and protein levels were also decreased in kidney tissue of hypertensive animals compared to control mice. Co-treatment of PMA and cinnamaldehyde also drastically inhibited the expression of pro-inflammatory cytokines Il-1β and Ccl2 compared to macrophages receiving only PMA. The application of cinnamaldehyde by itself did not induce Il-1β and Ccl2 expression, indicating that TRPA1 activation dampens pro-inflammatory macrophage activation but is unable to exert an effect by itself. However, cinnamaldehyde application did induce apoptosis of RAW267.4 macrophages, an effect that was decreased in the presence of HC-030031 [100]. A similar role for TRPA1 in macrophages was described in a different renal injury model, namely a renal ischemia-reperfusion injury (IRI) model. *Trpa1^-/-^* mice developed more severe symptoms of acute kidney injury and also had a higher influx of M1 macrophages in the kidneys in comparison to WT animals. In contrast to earlier reports, the number of M2 macrophages were unchanged between WT and *Trpa1^-/-^* mice. As described previously, the renal protein expression of TRPA1 was also decreased in WT animals following IRI compared to sham-treated mice. Although the levels of pro-inflammatory cytokines in *Trpa1^-/-^* mice were higher compared to WT animals, the anti-inflammatory cytokines Il-10 and TGF-β remained unchanged. These findings further confirm the anti-inflammatory role of TRPA1 by inhibiting the generation of M1 macrophages and concomitantly the production of inflammatory cytokines [101].

Although TRPA1 has a protective function in atherosclerosis and kidney injury, its implication in cardiac hypertrophy seems different. Mice, treated with a TRPA1 antagonist, showed attenuated cardiac hypertrophy, decreased expression of several hypertrophic markers (atrial natriuretic peptide, brain natriuretic peptide, and β-myosin heavy chain), and less interstitial fibrosis compared to untreated animals. Blocking TRPA1 also resulted in a decreased influx of M2 macrophages in the heart in comparison to untreated mice. Blocking TRPA1 also prevented the angiotensin II-induced expression M2 macrophage cytokines (Il-4, Il-10 and TGF-β). These results confirm that TRPA1 drives macrophage polarization towards an M2 phenotype and thereby potentially aggravates cardiac hypertrophy and fibrosis [102].

Inflammatory bowel disease (IBD) mainly affects the colon and the small intestine, causing symptoms including abdominal pain, diarrhea, and rectal bleeding. Since macrophages play an important role in the initiation and propagation of IBD [103], the anti-inflammatory role of TRPA1 in macrophages might be an interesting therapeutic target. Using a murine DDS-induced colitis model, TRPA1 was shown to be upregulated in DDS-treated mice and human IBD patient samples and was expressed in interstitial and infiltrating macrophages, respectively. *Trpa1^-/-^* DDS-treated mice showed more severe disease outcome, more severe histopathological alterations, increased TNFα expression levels, and higher Il-1β protein secretion compared to control mice. [84].

### 3.8. Dendritic Cells

#### 3.8.1. Expression

TRPA1 was not expressed in CD11c^+^ bone marrow-derived dendritic cells (BMDCs) [104,105]. It was, however, detected by qPCR in human monocyte-derived immature and mature DCs and, in immature DCs, also by immunofluorescence [88].

#### 3.8.2. (Patho)physiology

There have been no studies published on the function of TRPA1 in dendritic cells.

## 4. TRPA1 Expression and Function in Adaptive Immunity

### 4.1. T cells

#### 4.1.1. Expression

Experiments with qPCR, immunohistochemistry, patch clamp, and calcium imaging all showed that murine splenic CD4^+^ T cells express functional TRPA1 [104]. Later research confirmed the presence of TRPA1 in murine splenic CD3^+^ T cells using RT-PCR, immunohistochemistry, and flow cytometry [106]. TRPA1 was not detected in murine splenic Th2 lymphocytes via qPCR [57]. Immunohistochemistry analysis of the conjunctiva and cervical lymph nodes of mice with allergic conjunctivitis showed the expression of TRPA1 in CD4^+^ T cells [107]. TRPA1 was minimally expressed in murine skin CD3^+^ T cells [62], although immunohistochemistry revealed TRPA1 in dermal CD4^+^ T cells of inflamed skin samples [108].

TRPA1 was also detected in human peripheral blood mononuclear cell-derived T cells using immunohistochemistry and flow cytometry [106]. Human CD3^+^ T cells of the colon showed positive TRPA1 staining. Most of the TRPA1^+^ T cells were also TRPV1^+^ [104]. T cells in human oral submucosa did not show any TRPA1 immunostaining [92]. TRPA1 was also detected via Western Blot in Jurkat cells, an immortalized human T cell line [58].

#### 4.1.2. (Patho)physiology

Activation of T cells is a crucial part of the adaptive immune response. The T cell receptor (TCR)-induced Ca^2+^ response is a key step during T cell activation and helps to fine tune T cell fate [109]. Interestingly, pre-treating murine splenic T cells with two TRPA1 inhibitors, A967079 and HC-030031, completely abolished the TCR-induced Ca^2+^ responses. Moreover, the pharmacological inhibition of TRPA1 also diminished expression of the activation markers CD25 and CD69 upon T cell activation with CD3/CD28 or Concanavalin A [106]. These findings are in sharp contrast to earlier work by Bertin et al., in which *Trpa1^-/-^* CD4^+^ splenic T cells exhibited a higher and more sustained TCR-induced Ca^2+^ response. Interestingly, this effect on TCR-induced currents was significantly decreased following deletion or inhibition of TRPV1. Whole-cell perforated patch-clamp experiments also revealed increased capsaicin-induced currents in *Trpa1^-/-^* CD4^+^ T cells compared to WT cells. Although a compensatory mechanism in Bertin’s *Trpa1* KO mice could explain this discrepancy between the two studies, *Trpv1* mRNA and TRPV1 protein levels were the same in all mouse strains. These authors further explored the role of this regulatory TRPA1-TRPV1 interplay in a murine *Il10^-/-^* model of colitis. Histological analyses of the double KO animals showed increased signs of colonic inflammation and increased expression of proinflammatory cytokines in comparison with *Il10^-/-^* animals. CD4^+^ T cells from *Il10^-/-^Trpa1^-/-^* mice produced higher levels of the Th1 cytokines, IFN-γ and Il-2, following T cell activation. This effect was already observed before any symptoms of colitis had developed in these animals. TRPA1 knockdown in human CD4^+^ T cells also led to increased IFN-γ and Il-2 production, suggesting a conserved mechanism between human and mouse. These results suggest that TRPA1 restrains CD4^+^ T cell activation by diminishing TRPV1 channel function [104]. Interestingly, TRPV1/TRPA1 co-expression was not detected in dermal CD4+ T cells. This finding indicates that TRPA1/TRPV1 co-expression in CD4^+^ T cells, and their crosstalk, seems to be heavily dependent on tissue microenvironment [108].

The role of TRPA1 in other inflammatory conditions such as rheumatoid arthritis is much less clear. It was shown that the application of the sulphide donor, GYY4137, in a rodent serum-transfer arthritis model was able to ameliorate the arthritic symptoms in WT mice but not in *Trpa1* KO mice. This effect might have been caused by a combination of both neuronal and non-neuronal TRPA1. Because CD4^+^ T cells play a key role in the development of serum-transfer arthritis, T cell-mediated TRPA1-dependent effects cannot be included. Similar findings relate to neutrophils and macrophages, as the genetic removal of both these cell types protects against serum-transfer arthritis [66].

### 4.2. B Cells

#### 4.2.1. Expression

TRPA1 was detected via Western blot in Ramos cells, a Burkitt’s lymphoma cell line with B cell characteristics [58]. Immunohistochemistry analysis of human inflamed colon showed the presence of TRPA1 in infiltrating plasma cells [84].

#### 4.2.2. (Patho)physiology

There have been no studies published on the function of TRPA1 in B cells.

## 5. Conclusions

**Figure 3:**
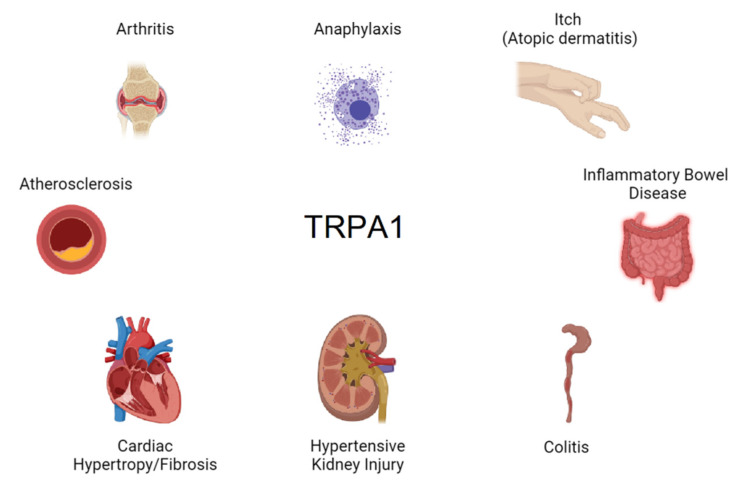
Pathologies linked to the expression of TRPA1 in immune cells.

By being expressed in nociceptive sensory nerve endings, epithelial cells, and immune system cells, TRPA1 sits at the first line of defense against tissue injury and invading pathogens. It is therefore no surprise that this channel is implicated in multiple inflammatory pathologies (Figure 3).

As known in nociceptive neurons [52], the ability of TRPA1 to be activated by signals associated with cell damage may also be crucial in the pathophysiology of immune cells. However, according to the existing literature, the contribution of this channel seems to be dual. On the one hand it may function as detector of cell stress, tissue injury, and exogenous noxious stimuli, leading to defensive responses; on the other hand, in conditions of aberrant regulation, TRPA1 may contribute to the exacerbation of inflammatory conditions. This is one reason that the future of TRPA1 as a therapeutic target in immune cells to remain unclear. The contradicting roles of TRPA1 in different disease models can be exemplified by the finding that TRPA1 activation induces M2 macrophage polarization [98,100,101,102]. However, M2 macrophages can have protective effects, such as in models of atherosclerosis [98] and kidney injury [100,101], but unfortunately, they were also shown to aggravate cardiac hypertrophy and fibrosis [102]. In this case, inhibition of TRPA1 would attenuate cardiac hypertrophy but increase atherosclerotic progression and worsen renal kidney injury. This problem could, in this specific case at least, be circumvented by excluding patients at risk for atherosclerosis and kidney injury from receiving TRPA1 inhibitors.

In addition, because of the wide expression pattern of this channel in neuronal and non-neuronal tissue, its inhibition was expected to be accompanied by adverse effects. Nonetheless, the only TRPA1 antagonist that has passed phase II clinical trials so far did not cause any significant serious side effects [110]. Unfortunately, development was later cancelled due to poor bioavailability and pharmacokinetics. Despite the lack of adverse effects, it is important to note that this study was performed over a period of only 4 weeks. Although there were no short-term adverse effects, long-term effects remain possible and should be further investigated during the development of future compounds. Additionally, the function of TRPA1 in non-neuronal cell types is still poorly understood and could also lead to unintended long-term effects of TRPA1 inhibition.

Unfortunately, a vast majority of studies in immune cells were restricted to document the gene and/or protein expression of TRPA1, whereas only a handful have assessed the channel functional properties (Table 1). Future studies should address this limitation, by, for instance, characterizing the biophysical and pharmacological properties and identifying the precise channel stimuli operating in each pathological condition, as essential steps in understanding the underlying mechanisms. Moreover, several human *TRPA1* gene variants have been associated with diseases, including familial episodic pain syndrome, cramp-fasciculation syndrome, asthma, and cough [111]. It would be thus interesting to determine how these variants may impact the function of immune cells.

## Figures and Tables

**Table 1:** Expression of TRPA1 in immune cells.

Cell Type	Subtype	TRPA1	Detection Method	References
Eosinophils	Murine skin eosinophils	No	Immunohistochemistry	[62]
	Human eosinophils nasal polyps	Yes	Immunohistochemistry	[63]
Neutrophils	Resting human neutrophils	Very low or absent	Microarray	[64]
Mast cells	BMMCs	Yes	RT-PCR	[69,70]
		Yes	Immunohistochemistry	[71]
		Yes	Western Blot	[71]
		Yes	Flow cytometry	[62]
		Yes	Ca2+ imaging via flow cytometry	[72]
		No	Ca2+ imaging	[73]
	Murine mast cells skin lesions	Yes	Immunohistochemistry	[62]
	Murine mast cell line C57.1	Yes	Ca2+ imaging via flow cytometry	[62]
	Rat RBL2H3 mast cell line	Yes	Western Blot	[74]
		Yes	Immunohistochemistry	[74]
	Human blood-derived mast cells	Yes	RT-PCR	[69]
	Human lung mast cells	No	Microarray	[75]
		No	Flow cytometry	[76]
	Human mast cells skin lesions	Yes	Immunohistochemistry	[62]
	Human mast cells nasal polyps	Yes	Immunohistochemistry	[63]
	Human mast cell line HMC-1	No	qPCR	[76]
		Weak staining	Flow cytometry	[76]
	Human mast cell line LAD2	No	Microarray	[75]
Macrophages	Naïve/activated murine peritoneal macrophages	No	qPCR	[81,82,83]
		No	Immunohistochemistry	[83]
		No	Ca2+ imaging	[83]
	Murine Langerhans cell	Weak staining	Immunohistochemistry	[62]
	Murine skin macrophages	Weak staining	Immunohistochemistry	[62]
	Murine interstitial macrophages	Yes	Immunohistochemistry	[84]
	Infiltrating murine macrophages atherosclerotic lesions	Yes	Immunohistochemistry	[85]
	Human alveolar macrophages (human lung parenchyma)	No	qPCR	[86]
	Human monocytes	Yes	qPCR	[87]
		Yes	Immunohistochemistry	[87]
		Yes	Western Blot	[87]
		No	qPCR	[61,88]
	Human alveolar macrophages	No	qPCR	[61]
	Human monocyte-derived macrophages	No	qPCR	[61]
	THP-1-derived macrophages	No	qPCR	[61]
		Yes	Immunohistochemistry	[89]
	U937-derived macrophages	No	qPCR	[61]
		Yes	qPCR	[90]
	Human infiltrating macrophages ectopic endometrial tissue	Yes	Immunohistochemistry	[91]
	Human infiltrating macrophages inflamed colon	Yes	Immunohistochemistry	[84]
	Human infiltrating macrophagesoral submucosa	Yes	Immunohistochemistry	[92]
	Human infiltrating macrophagesnasal polyps	Yes	Immunohistochemistry	[63]
	Human Langerhans cells	Yes	Immunohistochemistry	[58]
Dendritic cells	CD11c+ BMDCs	No	RT-PCR	[104]
		No	qPCR	[104,105]
	Human monocyte-derived immature DCs	Yes	qPCR	[88]
		Yes	Immunohistochemistry	[88]
	Human monocyte-derived mature DCs	Yes	qPCR	[88]
T cells	Murine splenic CD4+ T cells	Yes	qPCR	[104]
		Yes	Immunohistochemistry	[104]
		Yes	Ca2+ imaging	[104]
		Yes	Patch-clamp	[104]
	Murine splenic CD3+ T cells	Yes	RT-PCR	[106]
		Yes	Immunohistochemistry	[106]
		Yes	Flow cytometry	[106]
	Murine splenic Th2 cells	No	qPCR	[57]
	CD4+ T cells conjunctiva and cervical lymph nodes of mice with allergic conjunctivitis	Yes	Immunohistochemistry	[107]
	Murine Skin CD3+ T cells	Weak staining	Immunohistochemistry	[62]
	Murine dermal CD4+ T cells of inflamed skin samples	Yes	Immunohistochemistry	[108]
	Human blood-derived T cells	Yes	Immunohistochemistry	[106]
		Yes	Flow cytometry	[106]
	Human CD3+ T cells of the colon	Yes	Immunohistochemistry	[104]
	Human T cells oral submucosa	No	Immunohistochemistry	[92]
	Jurkat cells	Yes	Western blot	[58]
B cells	Ramos cells	Yes	Western blot	[58]
	Human infiltrating plasma cells inflamed colon	Yes	Immunohistochemistry	[84]

## Data Availability

This article contains no data.
